# GTI: A Novel Algorithm for Identifying Outlier Gene Expression Profiles from Integrated Microarray Datasets

**DOI:** 10.1371/journal.pone.0017259

**Published:** 2011-02-18

**Authors:** John Patrick Mpindi, Henri Sara, Saija Haapa-Paananen, Sami Kilpinen, Tommi Pisto, Elmar Bucher, Kalle Ojala, Kristiina Iljin, Paula Vainio, Mari Björkman, Santosh Gupta, Pekka Kohonen, Matthias Nees, Olli Kallioniemi

**Affiliations:** 1 FIMM - Institute of Molecular Medicine Finland, University of Helsinki, Helsinki, Finland; 2 Department of Pharmacology-Drug Development and Therapeutics, University of Turku, Turku, Finland; 3 Medical Biotechnology, VTT Technical Research Centre, Turku, Finland; National University of Ireland Galway, Ireland

## Abstract

**Background:**

Meta-analysis of gene expression microarray datasets presents significant challenges for statistical analysis. We developed and validated a new bioinformatic method for the identification of genes upregulated in subsets of samples of a given tumour type (‘outlier genes’), a hallmark of potential oncogenes.

**Methodology:**

A new statistical method (the gene tissue index, GTI) was developed by modifying and adapting algorithms originally developed for statistical problems in economics. We compared the potential of the GTI to detect outlier genes in meta-datasets with four previously defined statistical methods, COPA, the OS statistic, the t-test and ORT, using simulated data. We demonstrated that the GTI performed equally well to existing methods in a single study simulation. Next, we evaluated the performance of the GTI in the analysis of combined Affymetrix gene expression data from several published studies covering 392 normal samples of tissue from the central nervous system, 74 astrocytomas, and 353 glioblastomas. According to the results, the GTI was better able than most of the previous methods to identify known oncogenic outlier genes. In addition, the GTI identified 29 novel outlier genes in glioblastomas, including TYMS and CDKN2A. The over-expression of these genes was validated *in vivo* by immunohistochemical staining data from clinical glioblastoma samples. Immunohistochemical data were available for 65% (19 of 29) of these genes, and 17 of these 19 genes (90%) showed a typical outlier staining pattern. Furthermore, raltitrexed, a specific inhibitor of TYMS used in the therapy of tumour types other than glioblastoma, also effectively blocked cell proliferation in glioblastoma cell lines, thus highlighting this outlier gene candidate as a potential therapeutic target.

**Conclusions/Significance:**

Taken together, these results support the GTI as a novel approach to identify potential oncogene outliers and drug targets. The algorithm is implemented in an R package (Text S1).

## Introduction

The identification of genes associated with cancer development and progression is a central goal for many microarray data analysis projects [1]–[4]. Oligonucleotide microarrays offer clinicians and researchers the ability to analyze gene expression on a genome-wide scale. Expression arrays have been widely used in biological and clinical transcriptome studies for over a decade, and vast amounts of data have been accumulated in the public domain. For example, the Gene Expression Omnibus (GEO) database (http://www.ncbi.nlm.nih.gov/geo/) currently contains over 9247 expression studies in which human samples have been analyzed with gene expression microarrays [5].

Most microarray studies have focused on the identification of differentially expressed genes, using a panel of test and control samples collected at the same time and analyzed on a single platform. Most of these studies have been based on relatively homogeneous datasets consisting of comparably small numbers of samples. However, when results from such individual studies are compared with each other, the overlap of the differentially expressed gene sets is often minimal and disappointing. In order to identify consistently differentially expressed genes based on robust statistics, it is advisable to systematically combine multiple public datasets. The power of this ‘meta-analysis’ strategy has been demonstrated in the case of ArrayExpress [6], the Oncomine database [7], GeneSapiens [8], the Connectivity Map database [9] and several others. Large-scale integrated microarray datasets typically combine strongly diverging datasets based on different experimental conditions, independent cohorts of samples, varying sample preparation methods and labelling methods or scanner settings, and even different microarrays or microarray platforms. These multiple layers of variability pose a significant challenge to the statistical methods applied in meta-analyses. For example, the oligonucleotide array design utilized by Affymetrix, the leading manufacturer of expression arrays, has significantly changed over the last decade, resulting in many datasets with a variant probe set content and addressing variable numbers of genes. Several groups have already described methods for the integration of such diverse datasets [10], [11], [8]. As a result of these developments, there is a need for improved algorithms that facilitate the successful mining of heterogeneous multi-study or meta-analysis datasets.

Out of the many statistical methods used for the identification of differentially expressed genes [12], [13], the t-statistic has been one of the most basic and straightforward approaches for the analysis of individual studies. More recently, methods have been developed to detect differentially expressed genes in a subset of samples. These include cancer outlier profile analysis (COPA) [14], the outlier sum (OS) statistic [15] and the outlier robust t-statistic (ORT) [13]. COPA and OS statistics were derived from the t-statistic by replacing the mean and standard errors with the median and median absolute deviations, respectively. ORT was proposed as a more robust statistic that utilizes the absolute difference of each expression value from the median instead of the squared difference of each expression value from the average.

In general, outlier analysis offers a unique and powerful approach for the identification of key pathogenetic genes involved in a subset of disease samples. The strength of cancer outlier profile analysis was powerfully demonstrated by the identification of the TMPRSS2-ERG fusion oncogene in prostate cancers [14], considered a major breakthrough in cancer genetics. Another classic example of a typical cancer outlier gene is ERBB2/HER-2 [16], an important therapeutic target over-expressed in about 20% of human breast cancers. This is currently utilized for the therapy of HER2+ breast cancer patients with the therapeutic Herceptin antibody. Thus, genes generally expressed at low levels in normal samples, but over-expressed in a subset of cancer samples (although not all tumours), often represent potential drug targets of therapeutic interest, and may point to biologically different and diverse cancer subtypes that may require a specific form of individualized therapy.

**Figure 1:**
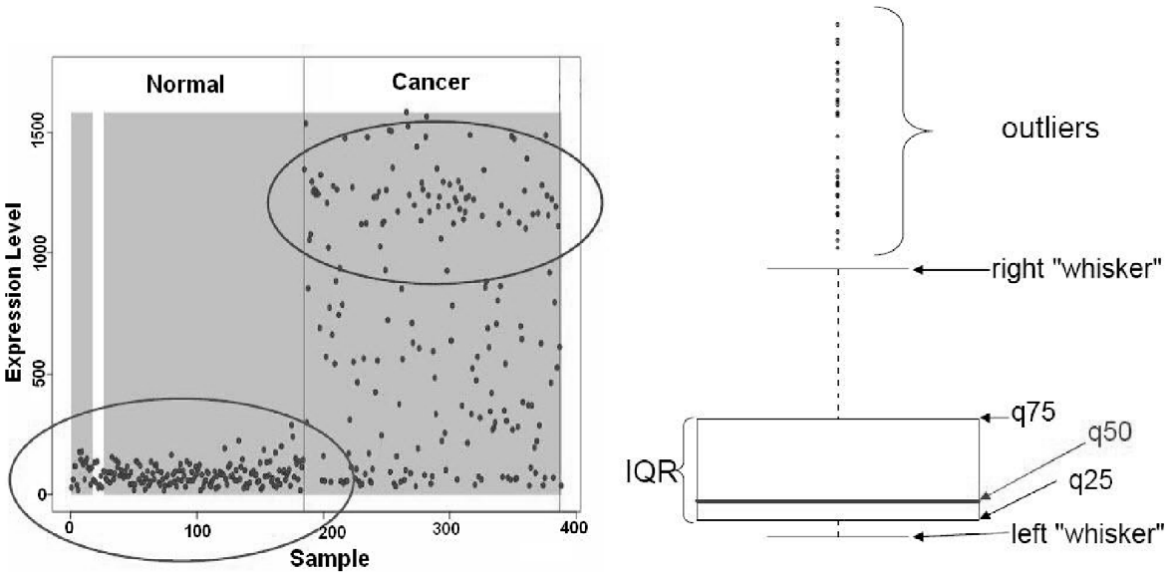
Illustration of a typical oncogene outlier profile. An example of a gene with high expression in the cancer group compared to the normal group. The circle in the cancer group refers to a subset of samples with high expression of this gene, while the circle in the normal group refers to a subset of normal samples, with low expression. The boxplot in B illustrates the concept of data outliers in standard terminology.

A gene showing over-expression in a subgroup of disease samples based on a cut-off threshold is defined as an outlier (Figure 1). Our aim was to find genes that are differentially expressed in a subset of test samples as compared to the controls. Here, we describe a novel statistical method for identifying genes with outlier expression in large-scale microarray data integration studies and compare this method with existing algorithms. These comparison methods include the t-statistic, cancer outlier profile analysis (COPA), the outlier sum (OS) statistic and outlier robust t-statistic (ORT).

COPA and OS statistics were derived from the t-statistic by replacing the mean and standard errors used in the t-statistic with the median and median absolute deviations, respectively. ORT has been proposed as a more robust statistic that utilizes the absolute difference of each expression value from the median instead of the squared difference of each expression value from the average.

In this study, we adapted an existing method from economics (the poverty index) with a comparable goal, addressing the question of how many people live below the poverty line in any given country, a formula developed for socioeconomic studies by Amartya Sen [17]. To adapt this algorithm for gene expression analysis, we inverted the original question here by asking, “in how many samples from the same body part is a gene X expressed above a fixed cut-off threshold?” Since the index is determined as a robust proportion of outlying samples, we assume that every gene is represented by an adequate number of samples. To this end, our aim was to establish an index that determines whether there is significantly increased gene expression in a sub-group of disease samples compared to the normal control group, without the restriction of making distribution assumptions for the various group populations. In preliminary studies, we observed that poverty indices derived from economics are well suited to measure the proportion of outlying samples within the disease sub-group relative to the reference group. Motivated by these observations, we modified the original poverty index formula [17], and in this paper we introduce the gene tissue index (GTI). The GTI is then systematically compared with the existing methods, i.e. t-statistics, COPA, OS and ORT. Furthermore, we compare the outlier detection capability of existing methods with the GTI using a simulated and real clinical large-scale integrated dataset. No comparative studies are currently available to support the suitability of the existing methods for the analysis of real, large-scale integrated meta-datasets such as those collected in the GeneSapiens[8] database.

## Materials and Methods

### Existing Statistical Methods

Let 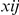 be the expression values for genes 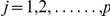 and samples 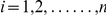. We assume that the gene expression samples are obtained from two different groups (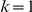 and 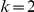), where 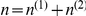. In our case, n(1) represents the number of samples from the normal group and n(2) represents the number of samples from the cancer group. Let Ck be the set of indices of the observations in group k, for k = 1 and 2.

### t-statistic

The formula for the standard unpaired t-statistic is:
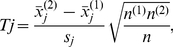
(2.1)where 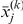 is the mean expression of samples for gene j in group *k*,
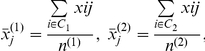


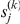
 is the pooled within-group standard deviation of gene *j*

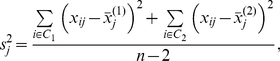
(2.2)assuming that the within-group standard deviations for the two groups are equal.

The two-sample t-test assumes that all disease samples for a particular gene are over-expressed. This assumption is not the case in cancer gene outlier analysis, where the genes are only assumed to be over-expressed in a subset of samples within the disease group that is assumed to be over-expressed [13].

### Cancer Outlier Profile Analysis (COPA)

The COPA [13], [14], [18]–[21] statistic is defined as the r^th^ percentile of the disease samples' standardized expression values 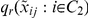, using r = 75, 90, or 95 as suggested by the authors. Observations for gene j are standardized by subtracting the median 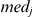 from each expression value (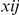) divided by the median absolute deviation 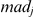


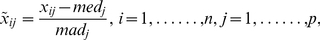
(2.3)where, 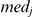 is the median and 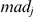 is the median absolute deviation of gene 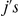 expression values.
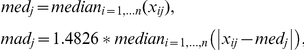
where the product of *mad_j_* and the constant 1.4826 is approximately equal to the standard error for normally distributed random variables.

The approach used in the COPA statistic addresses the problem of more accurately identifying genes with an outlier population than the t-statistic.

The COPA statistic is described as
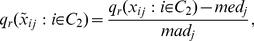
(2.4)where the r^th^ percentile of the disease samples is 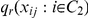.

Compared to the t-statistic, COPA intuitively replaces the normal sample mean by the all-sample median *med_j_*, the sample standard error s_j_ by the median absolute deviation *mad_j_*, and the disease sample mean by the r^th^ percentile 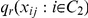.

It is evident that the COPA statistic may not be very robust, since a fixed r^th^ sample percentile is almost equal to using information from a single sample.

### Outlier Sums (OS)

The outlier sums statistic was introduced as an improvement over the COPA statistic. Here, the OS statistic [13], [15], [21] was proposed to replace the r^th^ percentile with a sum over the outlier samples from the disease group above a given cut-off. The OS statistic was designed to lower the false discovery rate (FDR) of COPA, as noted by Wu [13]. OS standardizes each expression value of gene j (x_ij_) through dividing the result of (x_ij_ – med_j_) by mad_j_. However, only expression values above a given cut-off are utilized for the final score.
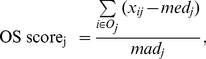
(2.5)where O_j_ is the set of outlier samples from the disease group defined by the following heuristic criterion:
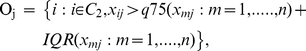
(2.6)where m refers to samples 1,2.....,n_1_,n_1_+1,....n.

### Outlier Robust t-statistic (ORT)

The outlier robust t-statistic[13], [19], [21] is a direct robust generalization of the two-sample t-statistic. With ORT, the sample mean is replaced with the median and the squared difference is replaced with the absolute difference. The overall median used as a common estimate for the two group medians was suggested to be inefficient, since the normal and disease samples are known to be different. The ORT statistic was therefore proposed to replace the overall median estimate used in calculating the COPA and OS score with a median calculated from the group median-centred expression values.
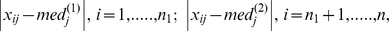
where 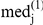 and 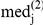 are the sample medians for normal and disease groups.
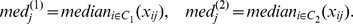



The median absolute deviation will then be estimated as
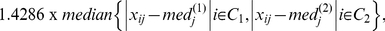
(2.7)which was proved to be similar to the pooled sample variance estimate
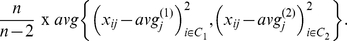



Here, the average (avg) is replaced with the sample median, and the squared difference is replaced with the absolute difference as a more robust estimate of the variance.

ORT is then described as
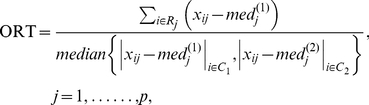
(2.8)where R is the set of outlier disease samples for gene j defined by
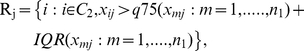
(2.9)where m refers to samples 1,2…..,n_1_.

It should be noted that only the normal group samples are used to estimate outliers when calculating an ORT score.

### Gene Tissue Index (GTI)

The GTI algorithm was originally used to calculate an index for a single treatment group k based on a standard cut-off. In this study, there was no defined standard cut-off per gene j for every normal tissue. Therefore, we defined a cut-off (B) based on the expression of gene j among all samples (n). These samples were obtained from one body part or tissue type such as the breast of normal (k = 1) and cancer-affected (k = 2) individuals. We then asked whether the proportion of samples above the cut-off is larger than it should be. Our choice of B is the standard statistical outlier cut-off (q75+IQR). We propose the following score, which weighs the proportion of outliers by a robust measure of how outlying the outliers are in a single group:
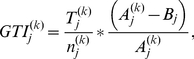
(2.10)where 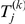 is the number of samples with expression values above the cut-off (number of elements in set 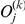), 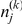 is the total number of samples in group k and 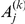 is the average expression of the samples above the cut-off for gene j.

We write
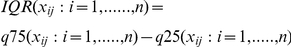
for the interquartile range (IQR).

Expanding the definition of the GTI and substituting our choice of B, we get
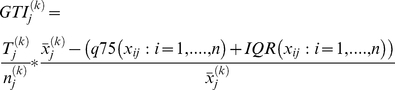
(2.11)where 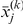 is the mean of ‘outlier samples’ in the group (k = 1 or k = 2) for genes 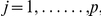


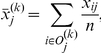
where the set 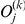 consists of the outliers in group k. The set 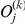 is defined using the following criterion:
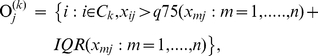
(2.12)where m refers to samples 1,2, …..,n(1),n(1)+1, …..n.

We calculate the actual GTI scores for each group k and gene j multiplied by 100, as this makes them more readable.

Finally, the index per gene is a direct association between two groups defined by 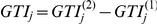, where 2 and 1 represent the grouping. The index GTIj can be a large positive number if there are outliers in the disease group 2 or a large negative number if there are outliers in the normal control group 1. All samples (cancer and normal combined) are then used to determine the cut-off point for each gene. As in the existing methods, we use permutations to estimate the null distribution and p-values of the GTI.

### Microarray data

The pre-processed Affymetrix transcriptome data utilized in this study were derived from the GeneSapiens database [8], and were acquired from multiple public repositories such as the Gene Expression Omnibus (GEO). In GeneSapiens, different Affymetrix array generations were normalized and combined to form a single large-scale multi-study dataset. It should be noted that the data in GeneSapiens are normalized first within a sample and then between samples using an Array Generation-based gene Centering (AGC) normalization [8]. The outlier analysis performed for the GTI evaluation study covered a total of 16 868 human genes, each represented by a different number of normal and cancer samples in the database. As the compositions of microarrays are regularly updated to incorporate new genes with improved target sequences, it is evident that combining data from different generations of the same microarray platform will generally result in largely varying numbers of samples per gene. We compared the log-transformed data of the normal group with the cancer group and performed five separate tests using the five methods introduced earlier.

### Cell culture and reagents

Human glioblastoma cell lines A172 and U87-MG were obtained from the ECACC (European Collection of Cell Cultures, UK), the LN-405 cell line was obtained from DSMZ (Deutsche sammlung von microorganismen und zellkulturen GmbH, Germany), and the U373-MG and astroglia SVG p12 cell lines from the ATCC (American Type Culture Collection, VA, USA). The A172, LN-405 and U373-MG cell lines were cultured in DMEM with 4500 mg/L glucose, 10% FBS, 2 mM L-glutamine and penicillin-streptomycin. U373-MG cells were supplemented with 300 ng/mL hygromycin. The U87-MG and SVGp12 cell lines were cultured in EMEM with 2 mM L-glutamine, 1 mM sodium pyruvate, 0.1 mM non-essential amino acids, 1.5 g/L sodium bicarbonate, penicillin-streptomycin and 10% FBS.

The antifolate drugs used in EC50 determinations were 5-fluorouracil (Sigma-Aldrich Co, St. Louis, Missouri), gemcitabine (trade name: Gemzar, Eli Lilly, Indianapolis, Indiana) and raltitrexed (trade name: Tomudex, Astra Zeneca, London, UK).

### Determination of the Median Effective Concentration (EC50)

The human glioblastoma cell lines A172 and U87-MG were obtained from the ECACC (European Collection of Cell Cultures, UK), LN-405 cell line was obtained from DSMZ (Deutsche Sammlung fuer Microorganismen und Zellkulturen GmbH, Germany), and the U373-MG and SVG p12 cell lines were purchased from the ATCC (American Type Culture Collection, VA, USA). The A172, LN-405 and U373-MG cell lines were cultured in DMEM with 4500 mg/L glucose, 10% FBS, 2 mM L-glutamine and penicillin-streptomycin. U373-MG cells were supplemented with 300 ng/mL hygromycin. The U87-MG and SVGp12 cell lines were cultured in EMEM with 2 mM L-glutamine, 1 mM sodium pyruvate, 0.1 mM non-essential amino acids, 1.5 g/L sodium bicarbonate, penicillin-streptomycin and 10% FBS. The antifolate drugs used in EC50 determinations were 5-fluorouracil (Sigma-Aldrich Co, St. Louis, Missouri), gemcitabine (trade name: Gemzar, Eli Lilly, Indianapolis, Indiana) and raltitrexed (trade name: Tomudex, Astra Zeneca, London, UK).

## Results

The suitability of existing outlier methods for the analysis of large-scale multi-study datasets is not only measured by the absolute statistical quality of the results obtained in theoretical settings, but strongly depends on a number of technological and practical issues. In this respect, it is mandatory to meticulously test whether such methods can be used for the analysis of extremely large-scale integrated microarray datasets. Currently, multi-study datasets such as those collected in GeneSapiens, or the clinical data sets provided by large-scale international cancer profiling consortia such as TCGA or ICGC, easily contain hundreds to several thousands of samples. The tendency towards large data sets will further increase with the progress of these integrated approaches, and with the introduction of next-generation genome sequencing technologies in cancer research. Some existing outlier methods may not be suitable to handle matrices with very many sample numbers, in particular if the data points available within these sets vary gene by gene. Even given the suitability of certain statistical approaches for successful outlier identification, the process may be exceedingly slow and may not be suitable for repeated application after every update to a database. For these reasons, we decided to evaluate the suitability of the GTI and the existing methods for identifying outliers in complex multi-study datasets.

### Comparison of the new GTI method with previously described outlier identification methods in a simulated single study dataset

First, to compare the GTI method with previously described outlier identification methods, we conducted simulation studies using ORT, OS, and COPA methods with a fixed statistical outlier cut-off (q75+IQR). In addition, the t-statistic was considered; however, this method did not require any cut-off selection. For the simulation, an artificial dataset was generated representing 1000 genes assuming an equal number of normal and cancer samples (*n* ^(1)^ = *n* ^(2)^ = 30), in which all expression values were drawn from a standard normal distribution. Next, we generated expression values for a gene assumed to be differentially expressed by adding a constant, *m*, to the expression values in only the first *k* cancer samples (k = 1, 10 or 30), where *k* equals the number of outlier samples, and used this value as the true positive (TP). The true positive and false positive (FP) values were calculated based on 50 simulations.

**Figure 2:**
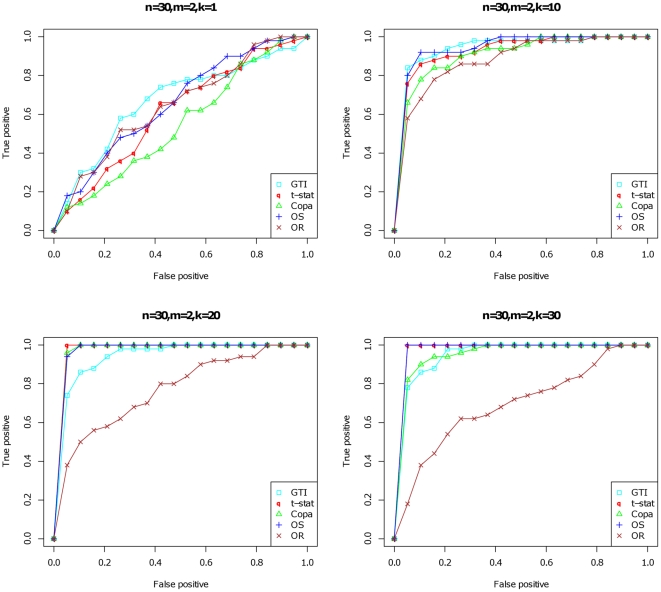
ROC curves for four different numbers of outliers. Receiver operating characteristic (ROC) curves from the above simulation study comparing the five statistics. One cancer gene is over-expressed by m units equal to 2 in k of the 30 samples. ROC curves are plotted based on 50 simulations. K refers to the number of outliers in the disease group.

In each simulation, a p-value was calculated as the proportion of genes with a score greater than that of the true positive. After collecting the 50 p-values, the true positive rate corresponding to a given false positive threshold was estimated as the proportion of simulations identifying the true positive gene using the false positive threshold. In other words, the generated p-value was not greater than the false positive rate. We varied the values of k (k = 30, 20, 10, 1) to simulate how the five statistical methods would perform in these different artificial cases. This procedure was repeated for each method as well as each dataset simulated with a varying value of k. The data from the simulations were used to calculate the true positive rate based on a given false positive threshold. These results are summarized in Table S1. The data in the Excel sheet named “ROC curve data from simulations” were used to construct the receiver operating characteristic (ROC) curves illustrated in Figure 2. Each curve generated for each method refers to a defined true positive rate for a particular method versus the false positive rate. An optimal method should generate large areas under the curve (AUC) for each simulated dataset.

The results presented in Figure 2 suggest that the GTI performs equally well to the other methods under single study settings. The first case of k = 1 (1 outlier in 30 samples) may exist, but is often neglected. The last case of having all samples in the disease group (k = 30) over-expressed is a typical profile for disease biomarkers rather than outlier expression.

The most interesting case of having k = 10 (10 outliers in 30 samples) showed that all methods were equally suited to identify the outliers. To visualize our approach, Figure S1 shows the simulated expression values of the top 12 genes ranked by the GTI. However, according to the results presented in Figure S1, all of these genes showed a strong outlier population in the cancer group.

Importantly, all of the simulations used to derive these results were carried out in a single study setting. However, multi-study data sets are becoming increasingly common and powerful resources, and it would be practical to test the applicability of these methods to multi-study datasets. To achieve this goal, our systematic analysis was extended by using pre-processed data from the GeneSapiens database.

### Application to a large-scale glioma microarray integrated dataset

**Figure 3:**
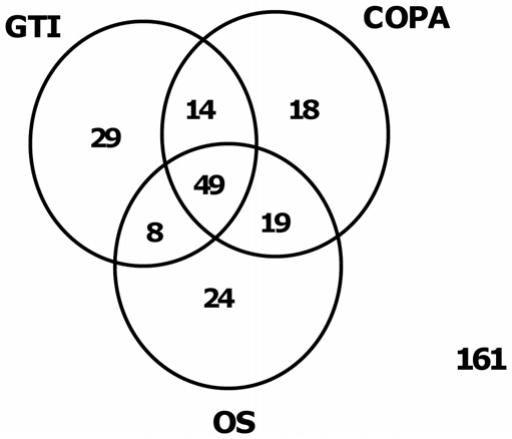
Venn diagram showing overlapping genes. The three sets of the top 100 genes only partially overlap, which means that at least one of them has many false positives or false negatives.

The GTI and the ORT, OS, and COPA methods were then tested with publicly available microarray datasets derived from central nervous system (CNS) tissues and tumours. We chose CNS tissue samples because there is an enormous wealth of data on glioblastoma in public repositories such as the Cancer Genome Atlas (TCGA) and GEO. Gliomas make up a group of primary CNS tumours that arise from glial cells. We focused on two subgroups of gliomas, anaplastic astrocytoma (WHO grade III) (74 samples) and glioblastoma multiforme (GBM, WHO grade IV) (353 samples). We used Affymetrix microarray data from healthy CNS tissues (392 samples) as a reference. The overlap of the top 100 genes identified by the GTI, COPA and the OS statistic is presented in a Venn diagram in Figure 3. The results indicate that approximately half of the genes could be identified by all three of these methods. COPA and the GTI showed more overlap than the GTI and OS; however, the OS statistic shared more genes with COPA than the GTI. The GTI identified 29 potentially novel outlier genes not identified in the top 100 by the other methods. Naturally, we wanted to focus on these 29 unique genes and further validate the GTI method by examining the value of these candidates in the cancer biology of glioma (see section on validation of targets below).

**Figure 4:**
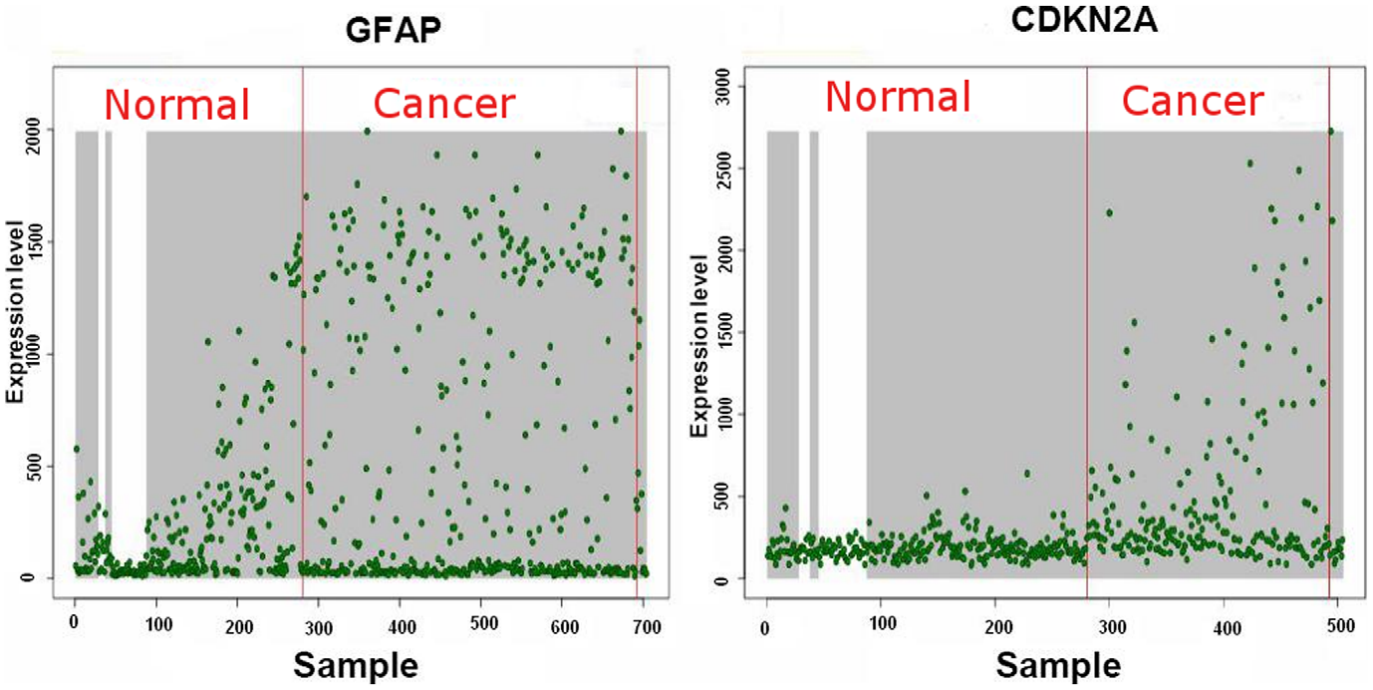
GeneSapiens database gene plots for GFAP and CDKN2A. The above plots are from the publicly available GeneSapiens database. The y-axis defines the expression level of each gene while the x-axis defines the number of samples per gene. We compared the expression of each gene among normal and cancer samples.

More specifically, we examined genes previously known to be over-expressed in and/or associated with the development, maintenance, and progression of glioblastoma multiforme and anaplastic astrocytoma and that were identified in the top 100 by any of the methods, as illustrated in Table 1. COPA identified seven known genes among the top 100 followed by the GTI (6), OS (4), ORT (3) and finally the t-statistic (2) (Table 1). All six genes identified by the GTI were also identified by COPA. More significantly, the GTI did not identify GFAP, a known differentiation marker for normal cells of astroglial origin as well as a glioma marker, which ranked highly using COPA (Table 1) [22], [23]. Next, we compared CDKN2A, which was the gene ranked lowest by the GTI (Table 1), with GFAP, which was only ranked highly by COPA. The distribution plots showed interesting differences between the respective gene expression profiles. While the gene expression levels of CDKN2A (ranked 71 with COPA and 92 with the GTI) showed a significantly clearer outlier population than GFAP (ranked 29 with COPA and 1866 with the GTI) (Figure 4), GFAP also showed a relatively high level of expression in the normal samples, indicating that this gene may in fact not be relevant for cancer progression. This pattern was similar for many of the genes uniquely identified by COPA, contradictory to its purpose of identifying outliers.

**Table 1:** Genes known to be associated with anaplastic astrocytoma and glioblastoma multiforme (GBM) out of the top 100 genes from every method after ranking according to the score.

Methods	Rank	Gene	GTI	COPA	OS	ORT	t-test
GTI	51	*VEGFA*		32	56	1	766
	**92**	***CDKN2A***		**71**	**212**	**15**	**2846**
	31	*EGFR*		33	125	41	1411
	32	*IL13RA2*		31	102	354	1664
	27	*IGFBP2*		4	10	3373	51
	65	*CHI3L1*		9	22	11962	405
COPA	32	*VEGFA*	51		56	1	766
	**71**	***CDKN2A***	**92**		**212**	**15**	**2846**
	33	*EGFR*	31		125	41	1411
	31	*IL13RA2*	32		102	354	1664
	4	*IGFBP2*	27		10	3373	51
	9	*CHI3L1*	65		22	11962	405
	**29**	***GFAP***	**1866**		**487**	**11990**	**6052**
OS	56	*VEGFA*	51	32		1	766
	10	*IGFBP2*	27	4		3373	51
	22	*CHI3L1*	65	9		11962	405
	71	*PDGFC*	1301	768		11968	24
ORT	1	*VEGFA*	51	32	56		766
	**15**	***CDKN2A***	**92**	**71**	**212**		**2846**
	41	*EGFR*	31	33	125		1411
t-test	51	*IGFBP2*	27	4	10	3373	
	24	*PDGFC*	1301	768	71	11968	

The rank column here refers to the position of the gene if all the 100 genes are sorted in descending order so that we have the first gene being the one with the highest outlier score for a particular method. Some of the methods, such as ORT, identified very few known outlier genes among the top 100.

To further examine the relevance of GFAP identified by COPA, but not the GTI, we also examined the expression of GFAP in tissue specimens. Specifically, immunohistochemical data from the Human Protein Atlas [www.proteinatlas.org] (HPA) confirmed that GFAP is expressed in all normal brain as well as brain cancer samples, and hence cannot be considered a ‘cancer outlier’ (data not shown). Briefly, and as illustrated by the GFAP mRNA expression profile, although not the only example, a high COPA score did not necessarily imply a significant outlier expression difference between the normal and cancer groups (Figure 4).

The observed results for GTI are to some extent in agreement with the genomic and transcriptional aberrations linked to gliomas. This is the case for some of the 14 genes presented in Table 2, which are known to be amplified or lost in significant subsets of gliomas. The ranks of selected oncogenes and tumour suppressor genes reported to be involved in glioblastoma multiforme (GBM) and anaplastic astrocytoma are presented in Table 2. It is apparent that some of the amplified and/or over-expressed genes, such as CDKN2A and CDKN2B, acquired high GTI scores, while commonly deleted genes such as PTEN acquired low scores. Interestingly, the GTI and COPA identified these over-expressed genes almost equally well, while all other methods (OS, ORT, t-statistics) performed significantly less well. The main difference between the GTI and COPA therefore appears to be the successful exclusion of false positive genes such as GFAP, which may be highly expressed in the glioma samples, but do not represent true outliers.

**Table 2:** Ranks of known anaplastic astrocytoma (grade III) and glioblastoma multiforme (GBM grade IV) genes.

No	Symbol	No. of PubMed refs	GTI	COPA	OS	ORT	t-test
**1**	***GFAP***	**965**	**1866**	**29**	**487**	**11990**	**6052**
2	*EGFR*	904	31	33	125	41	1411
3	*OLIG2*	44	3598	2773	13557	13643	5053
4	*CHI3L1*	19	65	9	22	11962	405
5	*IQGAP1*	6	1093	1141	1313	3314	1231
6	*IGFBP2*	61	27	4	10	3373	51
7	*IL13RA2*	2	32	31	102	354	1664
8	*MDM2*	169	7088	9138	6439	3623	7762
9	*RB1*	38	1869	2862	2621	2308	1788
**10**	***CDKN2A***	**367**	**92**	**71**	**212**	**15**	**2846**
11	*CDKN2B*	46	778	2790	418	244	3259
12	*PTEN*	471	5476	4524	2479	2557	3895
13	*TP53*	291	719	1325	1386	3342	709
14	*MCM3*	1	2673	3290	2837	1939	1484

The third column shows the number of articles as of 27/04/10 linking each gene to glioblastoma multiforme. Columns 4 to 8 present the rank of each gene among the five methods.

The glioblastoma large-scale integrated dataset used in this work contains varying numbers of samples per gene due to the combining of different generations of Affymetrix platforms. To predict whether the final score is affected by the varying number of samples, Spearman's rank correlation coefficient was calculated for each method from the scores of each method and the number of samples per gene (Table 3). A correlation value close to zero means that the final score of the method does not depend on the number of samples. As revealed in Table 3, OS and ORT statistics resulted in higher correlation values with the total number of samples, the number of normal samples and the number of cancer samples compared to GTI, the t-statistic and COPA.

**Table 3:** Spearman's rank correlation between the number of samples and ranks of genes per method.

	All samples	Count of normal samples	Count of cancer samples
GTI	−0.073	−0.056	−0.058
t-test	−0.059	−0.054	−0.072
COPA	0.042	0.022	0.069
OS	0.25	0.20	0.29
ORT	0.22	0.18	0.26

For each method, the scores are converted into ranks. Spearman's rank correlation is calculated between each method and the number of samples.

Overall, the GTI seemed to perform best in comparison to the other methods in identifying genes with an outlier profile among the glioma large-scale integrated dataset. Notably, when there are datasets with varying numbers of samples for different genes, the GTI, but not COPA, OS, ORT or the t-statistic, produced comparable scores among differentially expressed genes.

### Biological validation of some GTI top outliers

As stated above and shown in Table S2, 29 outlier genes were uniquely identified by the GTI (Table S2). Interestingly, among these 29, thymidylate synthase (TYMS) was the second best hit on the list (Table 4, GTI score 35, COPA score 105, OS score 1864, ORT score 1646), indicating high expression of this gene in many but not all glioblastomas, but generally low expression levels across the vast majority or all of the normal samples. GTI may therefore identify a putative subset of cancers that may particularly profit from therapy targeting the TYMS gene, which may not be the case for all of the glioblastoma patients.

**Table 4:** Assessing GTI-unique hits in relation to key terms (“glioblastoma” and “oncogene”) and their HPA staining images.

No	Symbol	No. of PubMed refs, keyword “glioblastoma”	No. of PubMed refs, keyword “oncogene”	Rank GTI	HPA outlier positive	HPA outlier negative	HPA outlier inconclusive
1.	LY96	1	4	29	**-**	**-**	**-**
2.	TYMS	0	5	37	✓		
3.	RASEF	0	0	42	✓		
4.	SRPX2	1	0	44	✓		
5.	VMO1	0	0	45	-	-	-
6.	TK1	5	8	52			✓
7.	CCNB2	2	50	57	✓		
8.	TREM1	2	3	58	✓		
9.	CHAC2	0	0	64	-	-	-
10.	CCDC102B	0	0	70	**-**	**-**	**-**
11.	VCAM1	0	4	71	✓		
12.	PRC1	2	39	72	✓		
13.	NCAPG	0	0	75	-	-	-
14.	NOD2	0	6	76	-	-	-
15.	NPW	0	4	79	-	-	-
16.	IL32	1	7	80	✓		
17.	GALK1	0	0	81	✓		
18.	EZH2	6	81	82	✓		
19.	WHSC1	2	11	84	✓		
20.	TTK	0	16	89	✓		
21.	TFPI	11	18	90	✓		
22.	PLEKHA4	0	0	91	-	-	-
23.	EHD2	0	0	93	-	-	-
24.	NMU	1	58	94	✓		
25.	C6orf173	0	0	95	-	-	-
26.	UBE2T	0	1	96	✓		
27.	PROM1	1	1	97	✓		
28.	ARSJ	0	0	99	✓		
29.	SOCS2	1	37	100			✓

Most of the 29 genes uniquely identified by the GTI have not been associated with oncogenic processes in glioblastoma, although there were several articles listed in PubMed linking them to oncogenic processes in other cancers as of 01/12/2010. The majority of the genes show variable immunohistochemical staining patterns in the Human Protein Atlas (HPA).

However, there are no publications to date linking TYMS to glioblastoma. In fact, only 7 of the 29 genes identified (24%) had been previously linked to glioblastoma in two or more publications (Table 4). Similarly, as many as 18 out of the 29 genes (62%) were linked to oncogenic functions in other cancers (Table 4). These observations suggest that the GTI identified more previously known oncogenes critical for the progression of other (e.g. epithelial) cancer types, which nevertheless have not been associated with glioblastoma. We also investigated protein staining images for 19 of the 29 genes uniquely identified by GTI, available in the human protein atlas database (HPA) (Table 4).

**Figure 5:**
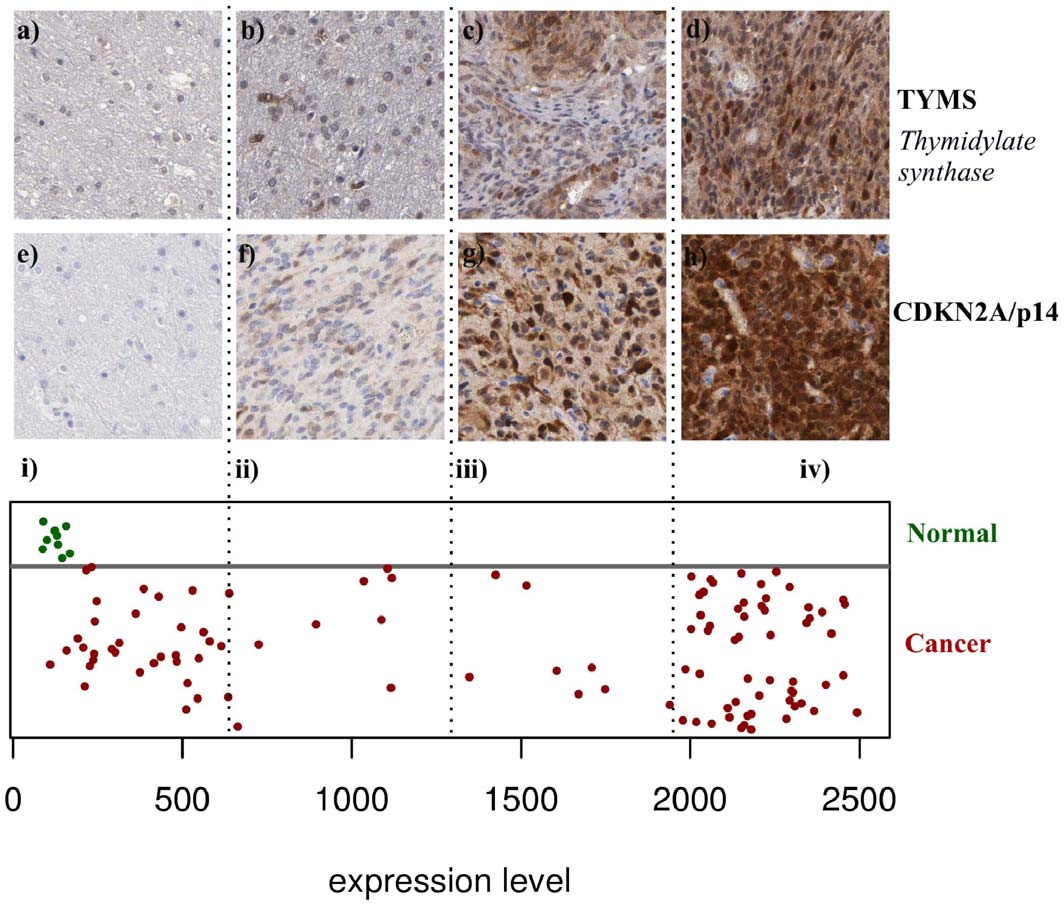
Human Protein Atlas images illustrating outlier gene expression. Glioma specimens were grouped into four categories of staining intensity (protein expression): i) negative, ii) weak, iii) moderate and iv) strong; a) and e) show staining of a normal sample. The upper panel TYMS/CDKN2A shows the staining of cancer samples for genes TYMS (b, c and d) and CDKN2A (f, g and h). Green-coloured samples represent normal tissue, and red-coloured samples represent cancer samples. In section (i) above containing (a) and (e), both normal and cancer samples show negative staining. The lower panel presents mRNA expression data divided into four quartiles illustrating a typical outlier pattern.

In contrast to the 29 unique GTI-identified candidates including TYMS, cyclin-dependent kinase inhibitor 2A, isoform 4/p16 (CDKN2A), was flagged by most of the methods (GTI score 92, COPA score 71, OS score 212, ORT score 15). This should therefore represent a cancer-relevant gene that is over-expressed in subsets of many different cancer types, but is not particularly specific for glioblastoma. For both CDKN2A and TYMS, immunohistochemical staining images were available from the HPA [24], [25], and were used to validate the outlier gene expression (Figure 5), assuming that differences in mRNA levels actually reflect differences in protein expression. Indeed, CDKN2A and TYMS showed strong positive staining in a subset, but not in all cancer samples (Figure 5). The Human Protein Atlas illustrated moderate or strong staining in 9 of 12 samples for TYMS and 5 out of 12 glioma samples for CDKN2A. For CDKN2A, 7 out of 12 glioma samples showed negative staining, possibly reflecting the fact that CDKN2A is often deleted in gliomas. Characteristic for a distinct outlier gene is the observation that one subgroup of samples showed very strong and uniform staining, while the remainder of the samples only showed very weak or negative staining, similar to the normal expression of this gene in non-malignant, control samples. Significantly, similar outlier protein staining patterns were observed in 17 out of the 19 genes (90%) for which a panel of immunostaining histology images was available in the HPA (Table 4).

TYMS is a known target of many antifolate drugs, such as 5′-fluorouracil (5FU) and gemcitabine. These drugs have been tested for the treatment of GBM patients, but have offered little general advantage over other treatment options [26]–[28]. Whereas 5FU and gemicitabine also inhibit RRM2 (ribonucleoside-diphosphate reductase M2 subunit) and DHFR (dihydrofolate reductase) [29], the new drug raltitrexed may be more specific to TYMS, but no published data are available for glioblastoma treatment.

**Figure 6:**
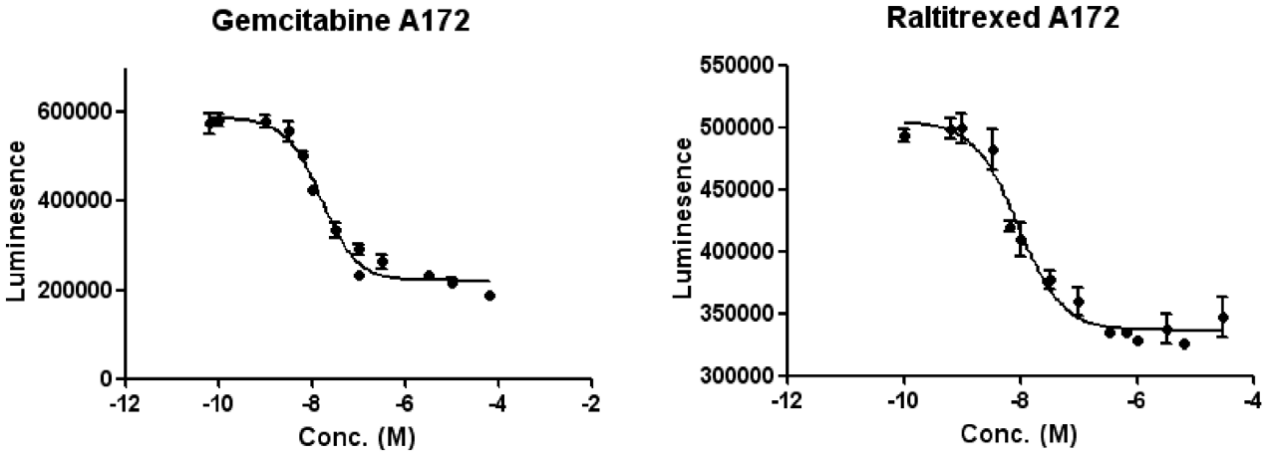
EC50 curves for raltitrexed and gemcitabine. EC50 curves show that the glioblastoma cell line A172 is more sensitive to raltitrexed than gemcitabine. EC50 values were determined using the Cell Titer Glo cell proliferation assay after four days of incubation with the drugs (see Text S1). Each data point and the standard deviations were calculated from six replicates.

Assuming that the concentration of the target gene would correlate with the sensitivity of cancer cells against the specific drug, we determined the median effective concentration (EC50) values for these three anti-folate drugs in cytotoxicity/cell proliferation assays with four established GBM cell lines and an immortalized foetal astrocyte cell line, SVGp12. Gemcitabine and the TYMS-specific raltitrexed showed EC50 values on the nM scale (Figure 6) against the GBM cell lines which all expressed significant levels of TYMS, whereas 5FU was only effective at 1000-fold higher concentrations (data not shown). The immortalized normal foetal astrocyte SVGp12, which only expressed TYMS at low levels, was 4- to 20-fold less sensitive to raltitrexed than any of the cancer cell lines. This suggests that TYMS may be a therapeutic target for glioma cells, and further studies to test the efficacy of a folate-targeting drug in gliomas showing outlier expression for TYMS should be undertaken.

Taken together, the results suggest that the detection of gene outliers with the GTI may provide complementary information on potential oncogenic genes in tumour tissue samples, and that these genes may be biologically relevant and associated with tumour progression.

## Discussion

In summary, we have shown how our newly-developed algorithm can be successfully utilized for the robust detection of outlier gene expression in cancers. The GTI performs particularly well with heterogeneous large-scale datasets that contain either variable numbers of samples between the groups being compared or variable numbers of informative samples for different genes. The GTI requires gene expression data from both disease tissues and corresponding reference samples, and approximates the proportion of the outlying population for each gene. Our results demonstrated that the GTI provided valuable complementary information when compared to existing analytical methods. This was demonstrated by the identification of 29 genes with outlier expression profiles not previously linked to glioblastoma. False positives, for example genes with high expression in both normal and cancer tissues, such as GFAP, [22], [23] were more often identified by the other methods such as COPA, which otherwise also performed well. With the increasing interest in examining outlier gene expression profiles in very large-scale meta-datasets, the GTI should complement the discovery of oncogenic genes and facilitate the identification of novel therapeutic targets.

When the proportion of outliers among the disease samples becomes very small, all existing methods become increasingly insensitive to the presence of small outlier populations. As demonstrated by Wu et al. (2007), COPA and the OS statistic use only cancer group samples to determine the rth percentile that is used as a cut-off to define outliers. Naturally, both of these methods therefore lead to the false-positive identification of general tissue-specificity marker genes such as GFAP, which also show high expression in the non-malignant, normal reference group. The approach of the GTI to utilize the normal control group for the calculation of outlier statistics therefore effectively reduces the number of false positives identified. Moreover, if the number of cancer samples is considerably larger than the number of normal samples (which is typical in most gene expression studies), COPA and OS and ORT statistics often show a significant increase in false positives (Figure 4). The ORT method only uses normal samples to determine the number of outliers [13], and did not perform well with any of the large-scale integrated datasets (Table 1 and 3). Importantly, we found that the GTI cut-off based on both cancer and normal samples significantly improved outlier analysis results. This improvement could explain why the GTI performed significantly better in large-scale integrated datasets.

We specifically explored the performance of COPA, OS, ORT, the t-statistic and the GTI in relation to the number of samples that were informative for a given gene. Importantly, the total number of informative samples introduces a strong bias in differential gene expression analysis, in particular among multi-study microarray datasets [30]. OS and ORT scores showed a correlation with the number of samples, pointing to such bias. The t-statistic and GTI were less affected by the number of samples (Table 3). For the COPA method, we found that most of the apparent false-positive hits among the top 100 had a large number of samples. The scaling factors used by older methods to achieve comparability of scores across different genes are not efficient when there are varying numbers of samples per gene present. This represents the major drawback of all of these methods, which does not affect the performance of the GTI. The use of the GTI is therefore particularly recommended in all instances where complex data structures and highly variable numbers of samples and data points per gene are provided. Furthermore, compared to other methods for outlier detection, our results clearly indicated that the scaling factor used for the GTI (T/N) efficiently dealt with the strong variation in samples per gene.

We validated the genes highlighted by the GTI using data from immunohistochemistry and explored their value as drug targets in glioblastoma cell lines. TYMS is a gene encoding thymidylate synthetase, which affects the production of nucleotide building-blocks used in DNA synthesis and repair. Thus, it is likely that fast growing and genetically unstable cancer cells are more dependent on TYMS function than normal cells. The immunhistochemical staining of the corresponding protein in HPA [24] demonstrated that TYMS is differentially expressed between normal and cancer samples (Figure 5), and indeed follows a characteristic outlier profile as predicted by the GTI. We also demonstrated that specific inhibitors of TYMS protein, such as raltitrexed, clearly showed cancer specificity, although we did not distinguish differential sensitivity among different tumour lines corresponding with the level of over-expression. Immortalized normal foetal astrocytes (SVGp12) were 4- to 2-fold less sensitive to raltitrexed than the four cancer cell lines tested, which all expressed similarly high levels of TYMS. We propose that the combination of outlier expression with knowledge of possible approved or experimental drugs based on information available from DrugBank [29] will open a possibility to reposition known drugs to cancer types for which they have not been previously used.

In order to better analyze heterogeneous large-scale microarray datasets, we developed the gene tissue index (GTI) as a new robust method for detecting cancer gene outliers. The GTI was used to examine over-expression and under-expression in cancer tissues as compared to a reference group. Our simulation results demonstrated that the GTI performs well as an alternative method for identifying outliers using single study datasets. Using a large-scale glioblastoma clinical dataset originating from multiple laboratories, the GTI identified more oncogene outliers than all of the previously described methods. Compared with most existing methods for outlier analysis, the GTI can uncover outliers by determining a cut-off based on all samples (both cancer and normal) and standardizing the score to achieve better comparability across genes. Most top-ranking genes according to the GTI showed a significant association with cancer.

## Supporting Information

Figure S1 **Expression profiles of GTI top ranking genes from the simulation study.** Plots of expression values in each group, for 12 genes ranked highest by the GTI statistic. Points have been jittered in the vertical direction for clear viewing. The blue colour refers to the normal and red to the cancer group. In the first plot with a GTI score of 6.906, it can be seen that the cancer group has a sub-population with expression above 9.
(PDF)

Table S1 Single study simulations data used to construct the receiver operating characteristic (ROC) curves.
(XLS)

Table S2 Twenty nine genes uniquely identified by GTI among the top 100 that were further followed up in this study.
(XLS)

Text S1 R programming code of the GTI algorithm introduced in this study used to identify cancer outliers.
(TXT)
