# Correction: GTI: A Novel Algorithm for Identifying Outlier Gene Expression Profiles from Integrated Microarray Datasets

**DOI:** 10.1371/annotation/7d571883-faf0-4f66-86a2-806c36c4741c

**Published:** 2011-04-05

**Authors:** John Patrick Mpindi, Henri Sara, Saija Haapa-Paananen, Sami Kilpinen, Tommi Pisto, Elmar Bucher, Kalle Ojala, Kristiina Iljin, Paula Vainio, Mari Björkman, Santosh Gupta, Pekka Kohonen, Matthias Nees, Olli Kallioniemi

The text under the subheading "Determination of the Median Effective Concentration (EC50)" is a repeat of the text in the section above it ("Cell culture and reagents."). The correct text is: Determination of the Median Effective Concentration (EC50) For each cell line 1500 cells per well were plated onto 384-well plates (Greiner Bio-One, Germany) 18 hrs before drug additions and then incubated in +37˚C, 5 % CO2. EC50 values were determined using CellTiter-Glo cell proliferation assay (Promega Corp, Madison, WI) after four day incubation with the drug dilutions. Six replicate wells were made for each drug dilution point and assay was repeated three times. Luminescence was measured with EnVision 2100 Multilabel platereader (PerkinElmer Inc., MA, USA). The EC50/IC50 values were calculated using sigmoidal dose response equation in GraphPad Prism version 5.0, GraphPad Software, San Diego, CA, USA.

