# Supplementary material for: GTI: A Novel Algorithm for Identifying Outlier Gene Expression Profiles from Integrated Microarray Datasets
Source: PLoS One. 2011 Feb 18;6(2):e17259. doi: 10.1371/journal.pone.0017259 (PMC3041823; doi:10.1371/journal.pone.0017259)
Supplement: Figure S1 — Expression profiles of GTI top ranking genes from the simulation study. Plots of expression values in each group, for 12 genes ranked highest by the GTI statistic. Points have been jittered in the vertical direction for clear viewing. The blue colour refers to the normal and red to the cancer group. In the first plot with a GTI score of 6.906, it can be seen that the cancer group has a sub-population with expression above 9. (PDF) [file pone.0017259.s001.pdf]

Figure S1

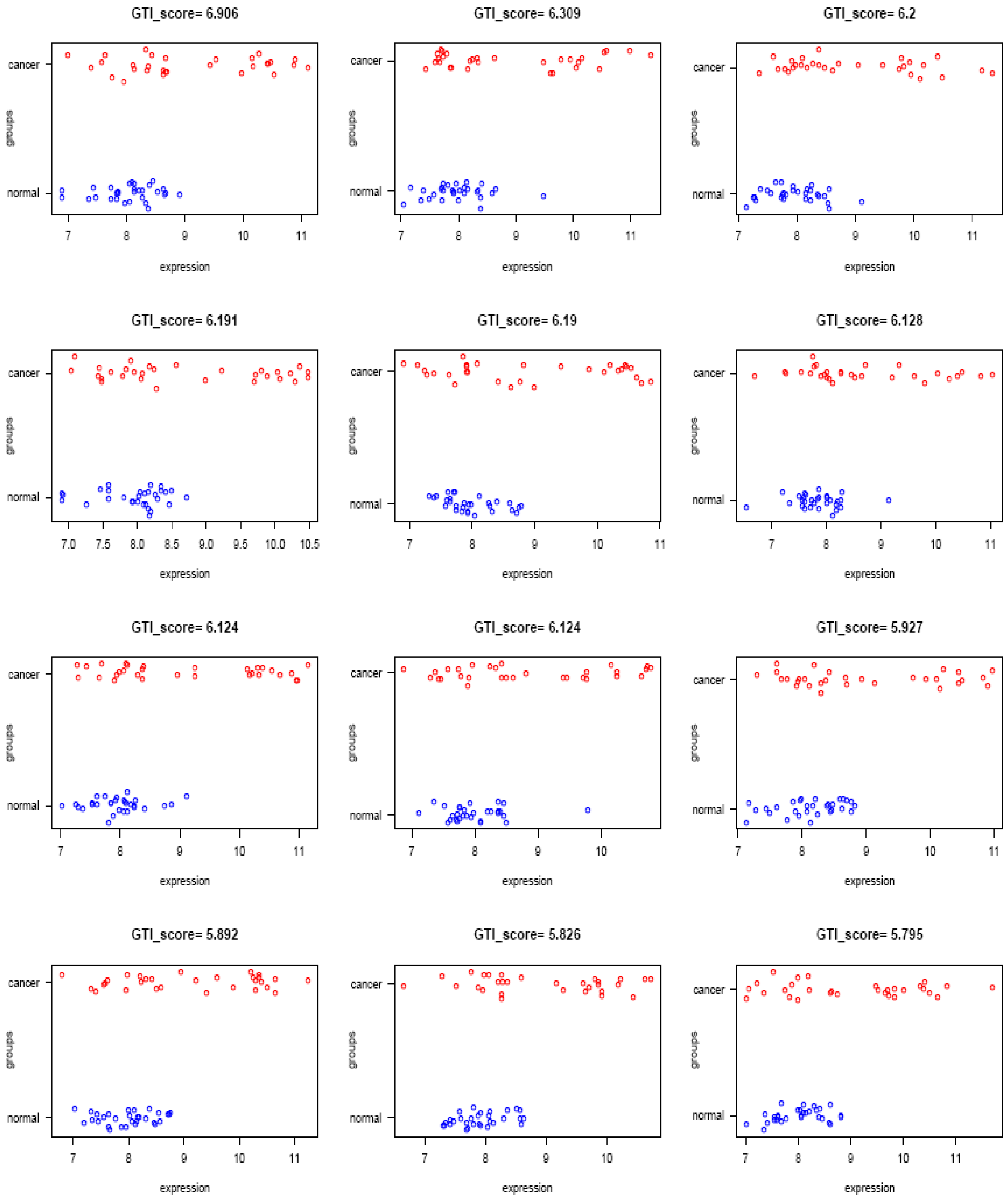

Fig. S1. Expression profiles of GTI top ranking genes from simulation study. Plots of expression values in each class, for 12 genes ranked highest by the GTI statistic. Points have been jittered in the vertical direction, for clear viewing. Blue color refers normal and red is the cancer group. In the first plot with GTI score = 6.906, it can be seen that the cancer group has a sub-population with expression above 9.
